# PPA1, an energy metabolism initiator, plays an important role in the progression of malignant tumors

**DOI:** 10.3389/fonc.2022.1012090

**Published:** 2022-11-25

**Authors:** Shuying Wang, Jianmei Wei, Shunwei Li, Yuyin Luo, Yifei Li, Xianglin Wang, Wenzhi Shen, Dehong Luo, Daishun Liu

**Affiliations:** ^1^ Department of Oncology, The Third Affiliated Hospital of Zunyi Medical University (The First People’s Hospital of Zunyi), Zunyi, China; ^2^ College of Clinical Medicine, Zunyi Medical University, Zunyi, China; ^3^ Department of Clinical Pharmacy, The Third Affiliated Hospital of Zunyi Medical University (The First People' s Hospital of Zunyi), Zunyi, China; ^4^ College of Clinical Medicine, Jining Medical University, Jining, China; ^5^ Key Laboratory of Precision Oncology in Universities of Shandong, Institute of Precision Medicine, Jining Medical University, Jining, China

**Keywords:** inorganic pyrophosphatase 1 (PPA1), tumor, biomarker, signaling pathways, epithelial-mesenchymal transition

## Abstract

Inorganic pyrophosphatase (PPA1) encoded by PPA1 gene belongs to Soluble Pyrophosphatases (PPase) family and is expressed widely in various tissues of Homo sapiens, as well as significantly in a variety of malignancies. The hydrolysis of inorganic pyrophosphate (PPi) to produce orthophosphate (Pi) not only dissipates the negative effects of PPi accumulation, but the energy released by this process also serves as a substitute for ATP. PPA1 is highly expressed in a variety of tumors and is involved in proliferation, invasion, and metastasis during tumor development, through the JNK/p53, Wnt/β-catenin, and PI3K/AKT/GSK-3β signaling pathways. Because of its remarkable role in tumor development, PPA1 may serve as a biological target for adjuvant therapy of tumor malignancies. Further, PPA1 is a potential biomarker to predict survival in patients with cancer, where the assessment of its transcriptional regulation can provide an in-depth understanding. Herein, we describe the signaling pathways through which PPA1 regulates malignant tumor progression and provide new insights to establish PPA1 as a biomarker for tumor diagnosis.

## Introduction

In 1926, Kay et al. identified a synthetic hydrolysis system in various human tissues and body fluids that balance the inorganic phosphates present in the body (1). In 1967, inorganic pyrophosphatase was first purified from human erythrocytes with a molecular weight of 42 KD (2, 3). The study of human-associated inorganic pyrophosphatases is gradually approaching maturity. In the past decades, proteomic analysis based on two-dimensional polyacrylamide gel electrophoresis and mass spectrometry has revealed that the expression of PPA1 is significantly increased in lung adenocarcinoma (4), primary colorectal carcinoma (5), infiltrating ductal carcinoma of the breast (6), prostate cancer (7), gastric cancer (8), liver cancer (9), large B-cell lymphoma (10), and ovarian cancer (11), compared with that in the corresponding normal or paraneoplastic tissues. It is significantly expressed in lung and breast cancer (12). PPA1, an energy-metabolizing enzyme, is encoded by a housekeeping gene and is widely expressed in various tissues of the body. PPA1 differential expression in normal tissues and corresponding malignant tumors indicates its potential as a molecular target for screening, diagnosing, and treating malignancies as well as predicting patient prognosis. Further studies have revealed that PPA1 is positively correlated with the progression of various malignant tumors as a result of its ability to facilitate tumor proliferation, suppress tumor apoptosis (12–14), and promote tumor metastasis by participating in epithelial-mesenchymal transition (EMT)-related signaling pathways (8, 15–17). In addition, a new human PPase, phospho-lysine phospho-histidine inorganic pyrophosphate phosphatase (LHPPase), has been cloned, and a significant increase of this protein was found to be associated with hyperthyroidism, while a decrease was observed in thyroid tumors (18).

Tumor cells are highly plastic and undergo rapid proliferation, invasion, and metastasis (19). The widespread expression of a protein such as PPA1 in tumor tissues and cells implies that it plays an extremely important role in the development of this malignancy. Based on this consideration, we describe the basic structure, function of PPA1 and the characteristics of its enzymatic activity, and summarize its role in malignancies with potential molecular mechanisms.

## Introduction of PPA1

### Properties and structure of PPA1

**Figure 1:**
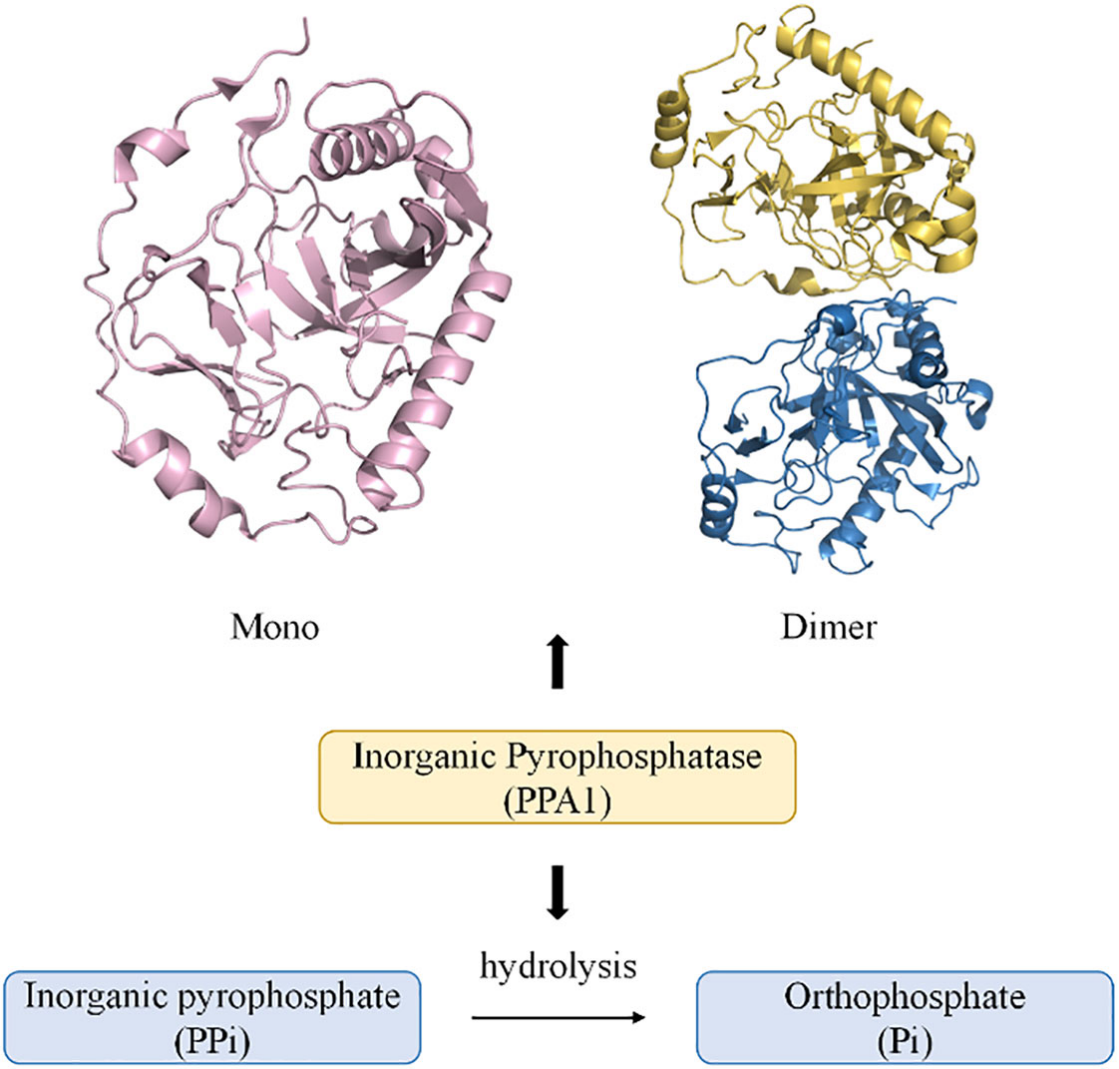
Diagram of the monomeric and dimeric structures of PPA1, and their modes of participation in PPi hydrolysis (21). (PDB code: mono 7BTN; dimer 7CMO).

PPases are often localized in the cytoplasm and are involved in the hydrolysis of PPi to form Pi. They also promote biological processes such as amino acid activation, nucleic acid polymerization, and nucleotide biosynthesis. Excessive accumulation of PPi can cause metabolic disorders in the body, leading to disease (3, 20) (**Figure 1**). A membrane-bound pyrophosphatase present in plants utilizes the energy of PPi hydrolysis for Na^+^ and H^+^ transport across the cell membrane (22). Further, a mitochondrial pyrophosphatase with catalytic subunits structurally and functionally similar to soluble PPases has also been reported (23–25). The three non-homologous families (I, II, and III) of soluble PPases display conserved functional elements with substantial overall sequence variation (26, 27). Family II pyrophosphatases (PPA2) in prokaryotes have poorly conserved protein residues, and family III pyrophosphatases (PPA3) are single structural domain proteins (20, 27). More closely associated with human organismal activity is the family I pyrophosphatase (PPA1), encoded by a housekeeping gene located on the long arm of chromosome 10 (28). Recent studies have shown that the crystal structure of human PPA1 can be determined at a resolution of 2.4 Å (29). It has a conserved dimeric structure that folds into a compact monomeric form with a molecular weight of 42 KD (3). The core is a substrate recognition site formed by a β-folded barrel-linked ring β5-β6, and the metal ion Mg^2+^ can bind to a binding groove near the β-folded barrel (20, 21, 29). The activity of PPA1 is closely associated with its function and is regulated by divalent cations. Such as free magnesium ions (Mg^2+^), it can stabilize PPase activity and act as a physiological activator (20, 30). The catalytic activity of PPase cannot be activated if there is a lack of divalent cations. The pH values also affect the hydrolytic activity of PPA1, with the highest activity at pH 6.5-7 (20). Pi, as an end product of the PPi hydrolysis, also inhibits the function of PPA1 to some extent (30). As an essential energy-metabolizing enzyme, PPA1 participates in various biosynthetic and metabolic pathways. Analyzing the differences between normal tissues and tumor tissues in terms of PPA1 enzymatic activity is beneficial to further investigate the potential role of PPA1 in the metabolic process for tumors. In a study by Shatton, J. B. et al., PPase activity was studied in a variety of rat tissues (31). The enzyme activity was significantly greater in liver and kidney tissues than in other tissues. Based on a per gram basis, PPase enzyme activity in liver is twice that of any other tissue at least, and 100 times greater than alkaline phosphatase activity, 13 times greater than glucose-6-phosphatase activity, and five times greater than ATPase activity. It is worth mentioning that the increase in enzyme activity in the tumor was pronounced (31). Furthermore, PPase activity is also affected by age and energy metabolism. Rats aged 24 months had a 2-fold greater liver activity of PPase than adult rats aged 4 months (32). PPase activity and expression increase in mice with short-term fasting, and refeeding reverse effect (33). Additionally, PPA1 has a self-assembly system that is dependent on the highly conserved amino acid residues Arg52 and Asp281. Nevertheless, PPA1 with mutated amino acid residues in self-assembly still exhibits enzymatic activity and promotes tumor cell growth, suggesting that self-assembly does not affect the biological function of PPA1 (21) (**Figure 1**).

### Biological functions of PPA1

PPA1 does not function solely as a hydrolase, but is also involved in biosynthetic functions through other metabolic mechanisms. Precursors of nicotinamide adenine dinucleotide (NAD^+^) are presented in all living cells which play a key role as coenzymes in the metabolism of substances and energy production. High levels of NAD^+^ contribute to the rapid proliferation of tumor cells (34). It is proved that silencing PPA1 inhibits NAD^+^ metabolism, leading to cell cycle arrest and cell death by autophagy in Baker’s yeast (35). Recently, the role of PPA1 in maintaining systemic metabolic stability has been explored. Mice deficient in the PPA1 gene fed a high-fat diet exhibited impaired glucose tolerance and severe insulin resistance, accompanied by impaired adipose tissue development and ectopic lipid accumulation. Mechanistic studies suggest that PPA1, a target gene of PPARc, maintains mitochondrial function in adipocytes (36).

During mammalian neuronal cell development, Tezuka et al. found that PPA1 over-expression in a mouse neuroblastoma cell line (N1E115) inhibited neurite growth after treatment with neuronal differentiation agents through dephosphorylation of phospho-c-Jun N-terminal kinase 1 (p-JNK1) (37). The effect of PPA1 on JNK dephosphorylation also induces type I collagen synthesis and stimulates calcification of osteoblasts (38). Furthermore, PPA1 has been shown to play a vital role in mediating tumor proliferation, apoptosis, and metastasis in a JNK activation-dependent or -independent manner; this is discussed in detail below (13, 14, 17).

## PPA1 promotes survival of malignant tumors

**Figure 2:**
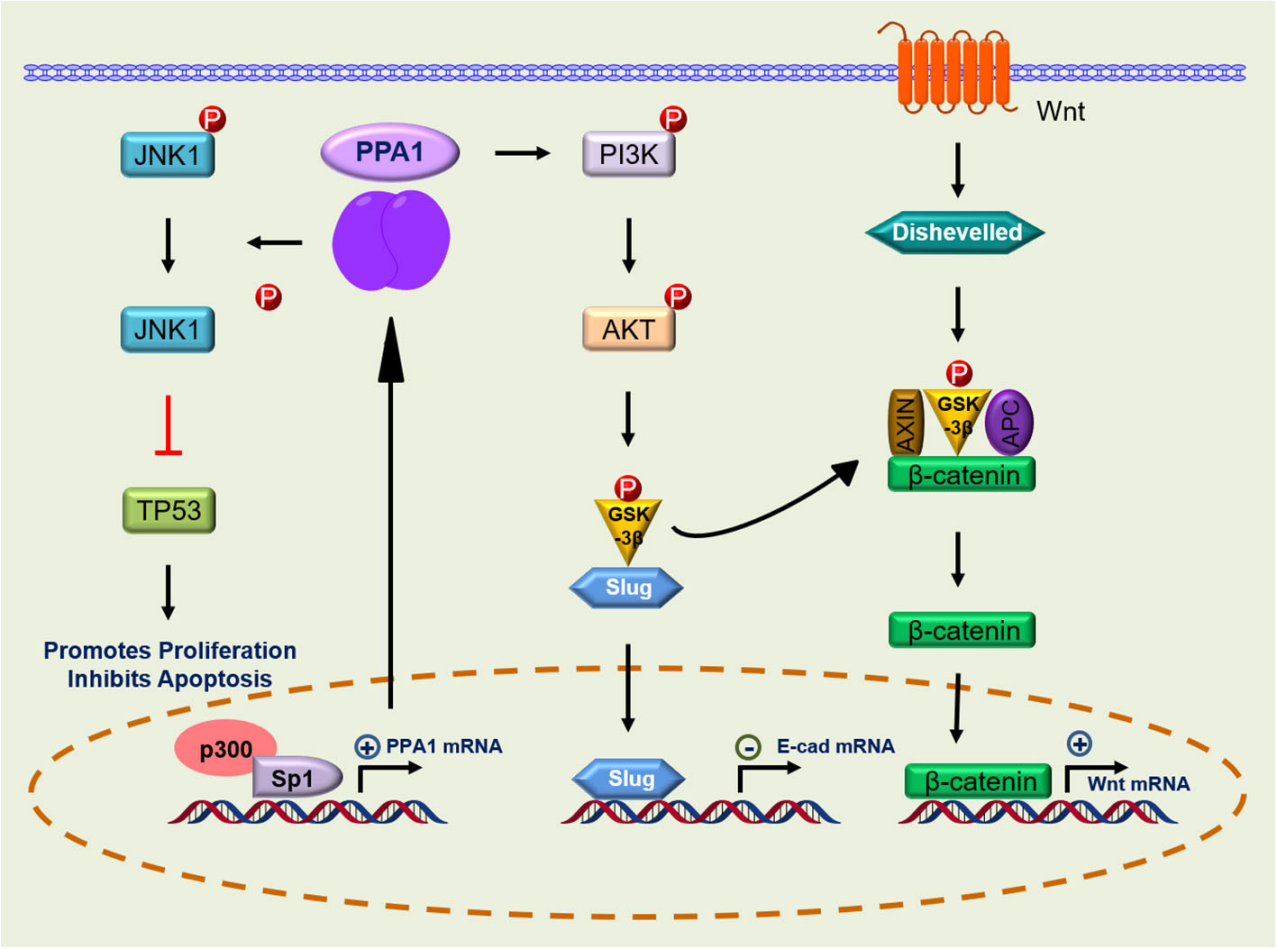
Signaling pathway of PPA1 in malignant tumor progression. PPA1, Inorganic pyrophosphatase; JNK-1, c-Jun N-terminal kinase 1; p53, p53 tumor suppressor homolog; p300, Histone acetyltransferase; Sp1, Sp1 transcription factor; PI3K, Phosphatidylinositol 3-kinase; AKT, AKT serine/threonine kinase; GSK-3β, glycogen synthase kinase 3 beta; Slug, snail family transcriptional repressor 2; Dishevelled, Dishevelled segment polarity protein 2 L homeolog; APC, APC regulator of WNT signaling pathway; Axin, Axin protein; p, Phosphorylation; ←, activation; ├, inhibition.

Owing to the extreme adaptability of malignancies, enhanced PPA1 expression suggests its requirement for tumor survival. In 2016, Luo et al. demonstrated that silence PPA1 *in vitro* reducing proliferation and promoting apoptosis in lung and breast cancer cells; the expression of cell cycle-related proteins p21 and p53 and cleaved caspase-3 was increased significantly, while the expression of proliferation-related protein Ki-67 was decreased (12). Similar findings were observed in diffuse large B-cell lymphoma (10), suggesting that the role of PPA1 in value-added apoptosis appears to be inextricably linked to p53. This was later confirmed in the lung cancer cell line H1299 (TP53 deficient), where silencing or overexpression of PPA1 did not affect the proliferation or apoptosis (12, 13). Wang et al. found that the proliferation and viability of colorectal cancer cells may be associated with upregulation of PPA1 and promotion of dephosphorylation of p-JNK1, while its expression did not affect the levels of p-ERK or p-p38 (14). Another study in lung cancer reported similar observations. Additionally, this significant increase in PPA1 expression inhibits apoptosis in lung cancer cells by dephosphorylating p-JNK1 at the peptide level (13) (**Figure 2**; **Table 1**).

**Table 1:** Role of inorganic pyrophosphatase (PPA1) in tumor progression.

Cancer type	Biological function of PPA1	Mechanism	Reference
Colon Cancer Lung Cancer	Promotes proliferation and inhibits apoptosis	JNK/P53	(13, 14)
Ovarian Cancer	Promotes tumor progression and increases cisplatin resistance	Circ_0067934/miR-545-3p/JNK	(39)
Human Epithelial Ovarian Cancer, EOC	Promotes proliferation and metastasis	Promotion of EMT by Wnt/β-catenin	(15)
Breast Cancer	Promotes proliferation and metastasis	Promoting EMT *via* PI3K/AKT/GSK-3β	(17)

Notably, expression of a pyrophosphatase active-inactivating mutant, PPA1 (D117A), abolished the PPA1-mediated apoptosis of the tumor, while inactivation of this active site also affected the dephosphorylation of p-JNK1 by PPA1 (13, 14). Whether PPA1 mediates tumor proliferation and apoptosis through dephosphorylation of JNK1 followed by regulation of p53 is not known, but some reports hint toward this possibility. Wang et al. eliminated the effect of PPA1 silencing on increased p53 expression levels using a JNK-specific inhibitor (SP600125) (14). Furthermore, microRNA (miR-545-3p) can target PPA1 to inhibit cell proliferation and invasion and enhance cisplatin resistance by increasing JNK phosphorylation in ovarian cancer (39) (**Table 1**).

## PPA1 promotes metastasis of malignant tumors

### PPA1 and EMT

The expression of PPA1 is significantly increased in the metastatic lymph nodes of malignancies, including gastric cancer (8, 40), colorectal cancer (14), ovarian cancer (16), and laryngeal squamous cell carcinoma (LSCC) (41) as assessed by immunohistochemistry or tissue microarrays, compared to that in controls. This means that PPA1 plays an essential role in the metastatic process of these tumors. Several functional experiments have confirmed this hypothesis. PPA1 over-expression in gastric cancer cell lines promotes its proliferation and increases its aggressiveness (8). Niu et al. studied the relationship between PPA1 and ovarian cancer tumorigenesis. They showed that PPA1 knockdown reduced the invasiveness and migration of ovarian cancer cells, and PPA1 expression was associated with EMT process. PPA1 silencing increases the expression of epithelial-specific marker E-cadherin and decreases the expression of mesenchymal-specific markers N-cadherin, vimentin, and smooth muscle actin (15). PPA1 also promotes the aggressiveness of tumor cells in ovarian cancer, and a positive correlation between β-catenin and PPA1 expression has been demonstrated (15, 16). In EMT process, tumor cells lose their ability to adhere and more easily metastasize *via* the blood or lymph to other locations (42, 43). Thus, PPA1 is most likely involved in tumor metastasis by promoting EMT.

### Regulation of EMT *via* Wnt/β-catenin signaling

The Wnt signaling pathway initiates intracellular signaling and plays an essential role in cell proliferation, differentiation, and tumor formation. β-catenin-T-cell factor (TCF)/lymphoid-enhancer factor is the hub of Wnt signaling pathway, and large amount of evidence suggest that it is involved in stemness, metabolic reprogramming, immune evasion, and therapeutic resistance of cancer cells (42, 43). Li et al. found that β-catenin expression was reduced after PPA1 silencing in ovarian serous carcinoma. In their work, total β-catenin, but not nuclear- or cytoplasm-derived β-catenin, were assayed, suggesting that PPA1 expression may play a role in the β-catenin signaling activation (16). An in-depth analysis of PPA1 showed that PPA1 silencing induces a slight reduction in the nuclear translocation of β-catenin, as well as a decrease in the transcriptional activity of TCF in the nucleus. In addition, EOC cell lines overexpressing PPA1 were treated with a series of Wnt/β-catenin specific inhibitors, in which the glycogen synthase kinase-3 beta (GSK-3β) inhibitor (KY021111) blocked the nuclear translocation of PPA1-promoted β-catenin (15). Nuclear translocation of β-catenin in the Wnt/β-catenin signaling pathway is a key process in Wnt activation. When GSK-3β phosphorylates β-catenin, it is hydrolyzed by intracytoplasmic proteases, resulting in the inability of intracellular β-catenin to accumulate and translocate to the nucleus to activate the corresponding transcription factors (44). The use of GSK-3β inhibitors blocks the process by which PPA1 promotes β-catenin nuclear translocation, implying that PPA1 promotes EMT in ovarian cancer by participating in β-catenin dephosphorylation (**Figure 2**; **Table 1**).

### Regulation of EMT *via* PI3K/AKT/GSK-3β signaling

The phosphatidylinositol 3-kinase (PI3K) and its downstream molecule AKT serine/threonine kinase (AKT), have been shown to be closely associated with tumor EMT, and activation of PI3K/AKT leads to the inhibition of epithelial characteristics and expression of mesenchymal proteins (45, 46). Guo et al. found that PPA1 acts as an activator of the PI3K/AKT/GSK-3β pathway and participates in the development of EMT induced by transcription factor Slug, thereby promoting breast cancer proliferation and metastasis (17). However, PPA1 is not directly upstream of PI3K, and their molecular interactors have not yet been reported. Elevated expression of p-PI3K (Tyr458) promotes phosphorylation of AKT (Ser473) and GSK-3β (Ser9) (17). Phosphorylated GSK-3β is degraded, releasing snail and β-catenin, which enter the nucleus to inhibit the transcriptional activity of E-cadherin (17, 47). Inhibition of E-cadherin during EMT causes epithelial cells to lose their ability to adhere and transform into a mesenchymal state (48). Slug, Twist, and zinc finger E-box-binding homeobox 1, which are transcription factors positively regulating the EMT program, were assessed and only Slug was found to be regulated by PPA1, where silencing PPA1 resulted in reduced Slug protein expression levels (17) (**Figure 2**; **Table 1**).

## PPA1, a biomarker for predicting survival prognosis

Analysis of several types of malignancies showed that PPA1 expression was closely correlated with clinicopathological staging. The higher the grade and stage of the tumor tissue, the higher the PPA1 expression. This has been observed in gastric cancer (8, 40), epithelial ovarian cancer (15), and colorectal cancer (14). The results of univariate and multivariate analyses have shown that PPA1 expression could also be used as a predictor of postoperative survival in clinical patients and as an independent predictor of overall survival (OS) (13, 14, 40, 49). PPA1 expression is significantly associated with intrahepatic cholangiocarcinoma (ICC) development, including tumor size, lymph node metastasis, differentiation, and TNM stage. Patients with PPA1-overexpressing tumors have reduced OS and higher recurrence rates than those with low PPA1 expression (49). More prominently, the expression of PPA1 is significantly higher in patients with advanced gastric cancer and in those with a poorer prognosis. However, there is no significant relationship between PPA1 expression and histological differentiation of gastric cancer (40). Overall, in malignancies with significantly increased PPA1 expression, PPA1 expression implies poor survival of patients.

## Transcriptional regulation of PPA1

Reports on the transcriptional regulation of PPA1 are scarce. In breast cancer cell line MCF7, Mishra et al. found three putative Sp1 binding sites in the promoter region of PPA1, which exhibited the highest transcriptional activity. Sp1 is a constitutive transcription factor located in the sequence of many housekeeping genes that play a regulatory role. It is overexpressed in many cancers and is associated with poor prognosis (50). Further validation showed that Sp1 activates PPA1 promoter activity, upregulates protein expression, and increases chromatin accessibility. Histone acetyltransferase (p300) activates the promoter activity of PPA1 induced by Sp1 (51). Notably, the CDK inhibitor (p16) expresses the key regulatory factor Sp1 which is required to maintain the activity of the proximal promoter necessary for p16 expression. This proximal promoter can also be modified by p300, which interacts directly with the reverse transcriptional activation domain of Sp1 and is recruited to the p16 promoter (50). Additionally, the PPA1 promoter may undergo local chromatin remodeling because of histone acetylation/deacetylation (51) (**Figure 2**). At the post-transcriptional level, PPA1 mRNA expression can be repressed by miR-545-3p, while circ_0067934, a molecular sponge of miR-545-3p, promotes the expression of PPA1 (39). It is expected that more miRNAs will be discovered and applied to clinical therapeutics targeting PPA1 in the future.

## Future perspectives

As the number of newly diagnosed cancer patients and cancer survivors continues to grow each year, it is tremendous pressure and burden on patients who are battling cancer and society at large (52, 53). It is an urgent need to discover effective biomarkers for diagnosis, treatment and prediction of patient survival. PPA1, an enzyme indispensable for maintaining energy metabolism, excels in the progression of several malignancies, regulating tumor cytogenesis development through the JNK/p53, Wnt/β-catenin and PI3K/AKT/GSK-3β signaling pathways. Based on this, we summarized the small molecule inhibitors (such as JNK-IN-8 (54), BKM120 (55) and Capivasertib (56)) targeting the above pathways for malignancy treatment, and found the feasibility and development potential of such therapeutic strategies (39, 57, 58). We also focused on PPA1 whose knockdown represses malignant abilities, such as tumor proliferation and migration. We noticed that designing small-molecule inhibitors to target PPA1 is a promising therapeutic strategy. To this end, designing molecular inhibitors of PPA1 or exploring more miRNAs to regulate PPA1 expression in malignant tumors, or combining with JNK (54) or PI3K-AKT inhibitors (55, 56, 59, 60) may be sensible choices.

**Figure 3:**
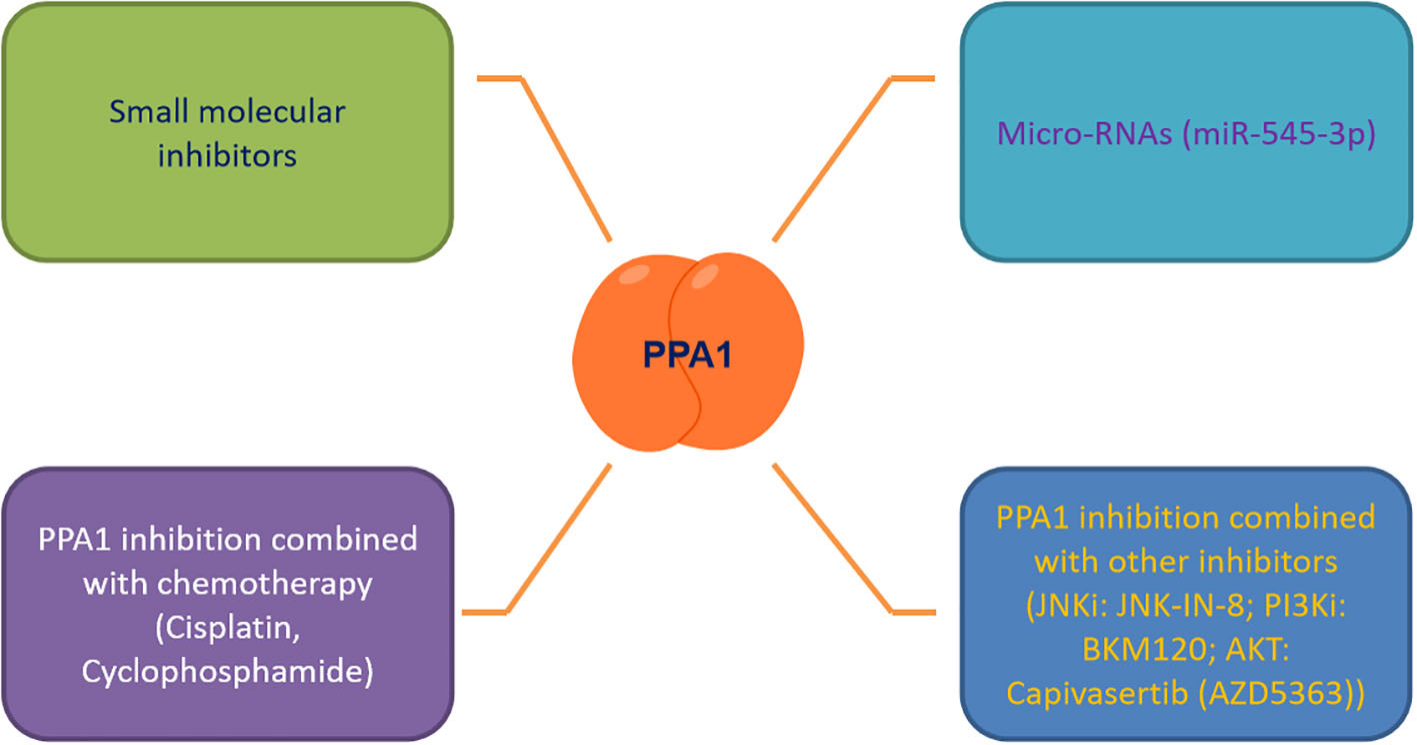
Targeting PPA1 to develop future therapeutic strategies.

Tumor microenvironment and metabolic reprogramming play a vital role in malignant tumor progression. PPA1, an energy metabolism-related enzyme, maintains the cellular metabolism in mitochondria and the expression of the key metabolite NAD^+^ (57, 61). Therefore, new therapeutic strategies targeting PPA1 need to be investigated, to curb the metabolic plasticity of tumors, either to be used as a standalone therapy or in combination with chemotherapy and other adjuvant therapies (**Figure 3**). We also propose that as PPA1 upregulation has been associated with high recurrence rates and low survival rates in patients with malignant tumors, PPA1 has substantial potential to be a reliable indicator of survival and prognosis in patients with tumors.

## Author contributions

SW conceptualized and wrote the original draft preparation. WS revised and reviewed the format. SL and YL assisted with the edited version. YFL and XW organized data and resources. J W performed visualization. DL and DHL assisted with the edited version and acquired the funding All authors contributed to the article and approved the submitted version.

## Funding

This study was supported by grants from Guizhou Provincial Natural Science Foundation Project ([2018]5623 to DL), Guizhou Provincial Respiratory Critical Disease Clinical Research and Prevention and Treatment Talent Base Project ([2020]8 to DL), Zunyi Respiratory Medicine Talent Base Project ([2019]69 to DL); Guizhou Science and Technology Cooperation Program ([2021] 456 to DHL); Research and Development Project of The First People’s Hospital of Zunyi ([2020] 11 to DHL); National Natural Science Foundation of China (No. 82173192 to WS).

## Conflict of interest

The authors declare that the research was conducted in the absence of any commercial or financial relationships that could be construed as a potential conflict of interest.

## Publisher’s note

All claims expressed in this article are solely those of the authors and do not necessarily represent those of their affiliated organizations, or those of the publisher, the editors and the reviewers. Any product that may be evaluated in this article, or claim that may be made by its manufacturer, is not guaranteed or endorsed by the publisher.

## References

[1] Kay HD . Kidney phosphatase. Biochem J (1926) 20(4):791–811. doi: 10.1042/bj0200791 16743720 PMC1251783

[2] Pynes GD Younathan ES . Purification and some properties of inorganic pyrophosphatase from human erythrocytes. J Biol Chem (1967) 242(9):2119–23. doi: 10.1016/S0021-9258(18)96026-6 6022858

[3] Thuillier L . Purification and kinetic properties of human erythrocyte Mg2+-dependent inorganic pyrophosphatase. Biochim Biophys Acta (1978) 524(1):198–206. doi: 10.1016/0005-2744(78)90118-3 656444

[4] Chen G Gharib TG Huang CC Thomas DG Shedden KA Taylor JM . Proteomic analysis of lung adenocarcinoma: identification of a highly expressed set of proteins in tumors. Clin Cancer Res (2002) 8(7):2298–305. doi: 10.1159/000064924 12114434

[5] Tomonaga T Matsushita K Yamaguchi S Oh-Ishi M Kodera Y Maeda T . Identification of altered protein expression and post-translational modifications in primary colorectal cancer by using agarose two-dimensional gel electrophoresis. Clin Cancer Res (2004) 10(6):2007–14. doi: 10.1158/1078-0432.CCR-03-0321 15041719

[6] Chahed K Kabbage M Ehret-Sabatier L Lemaitre-Guillier C Remadi S Hoebeke J . Expression of fibrinogen e-fragment and fibrin e-fragment is inhibited in the human infiltrating ductal carcinoma of the breast: the two-dimensional electrophoresis and MALDI-TOF-mass spectrometry analyses. Int J Oncol (2005) 27(5):1425–31. doi: 10.3892/ijo.27.5.1425 16211239

[7] Lexander H Palmberg C Auer G Hellström M Franzén B Jörnvall H . Proteomic analysis of protein expression in prostate cancer. Analytical quantitative cytol Histol (2005) 27(5):263–72. 16447818

[8] Jeong S-H Ko GH Cho YH Lee YJ Cho BI Ha WS . Pyrophosphatase overexpression is associated with cell migration, invasion, and poor prognosis in gastric cancer. Tumour Biol (2012) 33(6):1889–98. doi: 10.1007/s13277-012-0449-5 22797819

[9] Megger DA Bracht T Kohl M Ahrens M Naboulsi W Weber F . Proteomic differences between hepatocellular carcinoma and nontumorous liver tissue investigated by a combined gel-based and label-free quantitative proteomics study. Mol Cell Proteomics MCP (2013) 12(7):2006–20. doi: 10.1074/mcp.M113.028027 PMC3708182 23462207

[10] Li L Aruna, Luo D Jin A. . Clinical significance and functional validation of inorganic pyrophosphatase in diffuse large b cell lymphoma in humans. Cytotechnology (2018) 70(2):641–9. doi: 10.1007/s10616-017-0165-5 PMC5851958 29234945

[11] Wang L-N Tong SW Hu HD Ye F Li SL Ren H . Quantitative proteome analysis of ovarian cancer tissues using a iTRAQ approach. J Cell Biochem (2012) 113(12):3762–72. doi: 10.1002/jcb.24250 22807371

[12] Luo DH Wang GW Shen WZ Zhao ST Zhou W Wan L . Clinical significance and functional validation of PPA1 in various tumors. Cancer Med (2016) 5(10):2800–12. doi: 10.1002/cam4.894 PMC5083733 27666431

[13] Luo D Liu D Shi W Jiang H Liu W Zhang X . PPA1 promotes NSCLC progression via a JNK- and TP53-dependent manner. Oncogenesis (2019) 8(10):53. doi: 10.1038/s41389-019-0162-y 31551407 PMC6760234

[14] Wang P Zhou Y Mei Q Zhao J Huang L Fu Q . PPA1 regulates tumor malignant potential and clinical outcome of colon adenocarcinoma through JNK pathways. Oncotarget (2017) 8(35):58611–24. doi: 10.18632/oncotarget.17381 PMC5601679 28938583

[15] Niu H Zhou W Xu Y Yin Z Shen W Ye Z . Silencing PPA1 inhibits human epithelial ovarian cancer metastasis by suppressing the wnt/beta-catenin signaling pathway. Oncotarget (2017) 8(44):76266–78. doi: 10.18632/oncotarget.19346 PMC5652704 29100310

[16] Li H Xiao N Li Z Wang Q . Expression of inorganic pyrophosphatase (PPA1) correlates with poor prognosis of epithelial ovarian cancer. Tohoku J Exp Med (2017) 241(2):165–73. doi: 10.1620/tjem.241.165 28202851

[17] Guo C Li S Liang A Cui M Lou Y Wang H . PPA1 promotes breast cancer proliferation and metastasis through PI3K/AKT/GSK3β signaling pathway. Front In Cell Dev Biol (2021) 9:730558. doi: 10.3389/fcell.2021.730558 34595179 PMC8476924

[18] Koike E Toda S Yokoi F Izuhara K Koike N Itoh K . Expression of new human inorganic pyrophosphatase in thyroid diseases: Its intimate association with hyperthyroidism. Biochem Biophys Res Commun (2006) 341(3):691–6. doi: 10.1016/j.bbrc.2006.01.016 16430861

[19] Pe'er D Ogawa S Elhanani O Keren L Oliver TG Wedge D . Tumor heterogeneity. Cancer Cell (2021) 39(8):1015–7. doi: 10.1016/j.ccell.2021.07.009 34375606

[20] Baykov AA Cooperman BS Goldman A Lahti R . Cytoplasmic inorganic pyrophosphatase. Prog Mol subcell Biol (1999) 23:127–50. doi: 10.1007/978-3-642-58444-2_7 10448675

[21] Hu F Huang Z Zheng S Wu Q Chen Y Lin H . Structural and biochemical characterization of inorganic pyrophosphatase from homo sapiens. Biochem Biophys Res Commun (2020) 533(4):1115–21. doi: 10.1016/j.bbrc.2020.09.139 33036755

[22] Tsai J-Y Kellosalo J Sun YJ Goldman A . Proton/sodium pumping pyrophosphatases: the last of the primary ion pumps. Curr Opin In Struct Biol (2014) 27:38–47. doi: 10.1016/j.sbi.2014.03.007 24768824

[23] Volk SE Baykov AA Kostenko EB Avaeva SM . Isolation, subunit structure and localization of inorganic pyrophosphatase of heart and liver mitochondria. Biochim Et Biophys Acta (1983) 744(2):127–34. doi: 10.1016/0167-4838(83)90081-X 6132623

[24] Lundin M Baltscheffsky H Ronne H . Yeast PPA2 gene encodes a mitochondrial inorganic pyrophosphatase that is essential for mitochondrial function. J Biol Chem (1991) 266(19):12168–72. doi: 10.1016/S0021-9258(18)98875-7 1648084

[25] Kajander T Kellosalo J Goldman A . Inorganic pyrophosphatases: one substrate, three mechanisms. FEBS Lett (2013) 587(13):1863–9. doi: 10.1016/j.febslet.2013.05.003 23684653

[26] Baykov AA Anashkin VA Salminen A Lahti R . Inorganic pyrophosphatases of family II-two decades after their discovery. FEBS Lett (2017) 591(20):3225–34. doi: 10.1002/1873-3468.12877 28986979

[27] Anashkin VA Aksenova VA Salminen A Lahti R Baykov AA . Cooperativity in catalysis by canonical family II pyrophosphatases. Biochem Biophys Res Commun (2019) 517(2):266–71. doi: 10.1016/j.bbrc.2019.07.056 31349973

[28] Fairchild TA Patejunas G . Cloning and expression profile of human inorganic pyrophosphatase. Biochim Biophys Acta (1999) 1447(2-3):133–6. doi: 10.1016/S0167-4781(99)00175-X 10542310

[29] Niu H Zhu J Qu Q Zhou X Huang X Du Z . Crystallographic and modeling study of the human PPA1 (*Inorganic pyrophosphatase* 1): A potential anti-cancer drug target. Proteins (2021) 89(7):853–65. doi: 10.1002/prot.26064 33583053

[30] Josse J . Constitutive inorganic pyrophosphatase of escherichia coli. 1. purification and catalytic properties. J Biol Chem (1966) 241(9):1938–47. doi: 10.1016/S0021-9258(18)96650-0 5329747

[31] Shatton JB Shah H Williams A Morris HP Weinhouse S . Activities and properties of inorganic pyrophosphate in normal tissues and hepatic tumors of the rat. Cancer Res (1981) 41(5):1866–72. doi: 10.1016/0304-3835(81)90102-6 6111393

[32] Panda H Pandey RS Debata PR Supakar PC . Age-dependent differential expression and activity of rat liver cytosolic inorganic pyrophosphatase gene. Biogerontology (2007) 8(5):517–25. doi: 10.1007/s10522-007-9094-6 17415680

[33] Kharbhih WJ Sharma R . Age-dependent increased expression and activity of inorganic pyrophosphatase in the liver of male mice and its further enhancement with short- and long-term dietary restriction. Biogerontology (2014) 15(1):81–6. doi: 10.1007/s10522-013-9481-0 24271717

[34] Navas LE Carnero A . Nicotinamide adenine dinucleotide (NAD) metabolism as a relevant target in cancer. Cells (2022) 11(17):2627. doi: 10.3390/cells11172627 36078035 PMC9454445

[35] Serrano-Bueno G Hernandez A Lopez-Lluch G Perez-Castineira JR Navas P Serrano A . Inorganic pyrophosphatase defects lead to cell cycle arrest and autophagic cell death through NAD+ depletion in fermenting yeast. J Biol Chem (2013) 288(18):13082–92. doi: 10.1074/jbcM112439349 PMC3642350 23479727

[36] Yin Y Wu Y Zhang X Zhu Y Sun Y Yu J . PPA1 regulates systemic insulin sensitivity by maintaining adipocyte mitochondria function as a novel PPARγ target gene. Diabetes (2021) 70(6):1278–91. doi: 10.2337/db20-0622 33722839

[37] Tezuka Y Okada M Tada Y Yamauchi J Nishigori H Sanbec A . Regulation of neurite growth by inorganic pyrophosphatase 1 via JNK dephosphorylation. PloS One (2013) 8(4):e61649. doi: 10.1371/journal.pone.0061649 23626709 PMC3633968

[38] Polewski MD Johnson KA Foster M Millán JL Terkeltaub R . Inorganic pyrophosphatase induces type I collagen in osteoblasts. Bone (2010) 46(1):81–90. doi: 10.1016/j.bone.2009.08.055 19733704 PMC2818162

[39] Yin Y Li J Rong J Zhang B Wang X Han H . Circ_0067934 reduces JNK phosphorylation through a microRNA-545-3p/PPA1 axis to enhance tumorigenesis and cisplatin resistance in ovarian cancer. Immunopharmacol Immunotoxicol (2022) 44(2):261–74. doi: 10.1080/08923973.2022.2038193 35179434

[40] Yang Y Cai J Yin J Wang D Bai Z Zhang J . Inorganic pyrophosphatase (PPA1) is a negative prognostic marker for human gastric cancer. Int J Clin Exp Pathol (2015) 8(10):12482–90. PMC4680380 26722435

[41] Cai G-M Huang DH Dai YZ Liu Y Pi LM Tan HL . Analysis of transcriptional factors and regulation networks in laryngeal squamous cell carcinoma patients with lymph node metastasis. J Proteome Res (2012) 11(2):1100–7. doi: 10.1021/pr200831g 22070577

[42] Manfioletti G Fedele M . Epithelial-mesenchymal transition (EMT) 2021. Int J Mol Sci (2022) 23(10):5854. doi: 10.3390/ijms23105848 35628655 PMC9145979

[43] Brabletz S Schuhwerk H Brabletz T Stemmler MP . Dynamic EMT: a multi-tool for tumor progression. EMBO J (2021) 40(18):e108647. doi: 10.15252/embj.2021108647 34459003 PMC8441439

[44] Nusse R Clevers H . Wnt/β-catenin signaling, disease, and emerging therapeutic modalities. Cell (2017) 169(6):985–99. doi: 10.1016/j.cell.2017.05.016 28575679

[45] Xu W Yang Z Lu N . A new role for the PI3K/Akt signaling pathway in the epithelial-mesenchymal transition. Cell Adhesion Migration (2015) 9(4):317–24. doi: 10.1080/19336918.2015.1016686 PMC4594353 26241004

[46] Hinz N Jücker M . Distinct functions of AKT isoforms in breast cancer: A comprehensive review. Cell Communication Signaling CCS (2019) 17(1):154. doi: 10.1186/s12964-019-0450-3 31752925 PMC6873690

[47] Lin J Song T Li C Mao W . GSK-3β in DNA repair, apoptosis, and resistance of chemotherapy, radiotherapy of cancer. Biochim Et Biophys Acta Mol Cell Res (2020) 1867(5):118659. doi: 10.1016/jbbamcr2020118659 31978503

[48] Wang Y Shi J Chai K Ying X Zhou BP . The role of snail in EMT and tumorigenesis. Curr Cancer Drug Targets (2013) 13(9):963–72. doi: 10.2174/15680096113136660102 PMC4004763 24168186

[49] Xu D Miao Y Gu X Wang J Yu G . Pyrophosphatase 1 expression is associated with future recurrence and overall survival in Chinese patients with intrahepatic cholangiocarcinoma. Oncol Lett (2018) 15(5):8095–101. doi: 10.3892/ol.2018.8278 PMC5934715 29740496

[50] Beishline K Azizkhan-Clifford J . Sp1 and the 'hallmarks of cancer'. FEBS J (2015) 282(2):224–58. doi: 10.1111/febs.13148 25393971

[51] Mishra DR Chaudhary S Krishna BM Mishra SK . Identification of critical elements for regulation of inorganic pyrophosphatase (PPA1) in MCF7 breast cancer cells. PloS One (2015) 10(4):e0124864. doi: 10.1371/journal.pone.0124864 25923237 PMC4414593

[52] DeSantis CE Lin CC Mariotto AB Siegel RL Stein KD Kramer JL . Cancer treatment and survivorship statistics. CA: Cancer J Clin (2014) 64(4):252–71. doi: 10.3322/caac.21235 24890451

[53] Weir HK Thompson TD Soman A Moller B Leadbetter S White MC . Meeting the healthy people 2020 objectives to reduce cancer mortality. Prev Chronic Dis (2015) 12:E104. doi: 10.5888/pcd12.140482 26133647 PMC4492213

[54] Soleimani M Somma A Kaoud T Goyal R Bustamante J Wylie DC . Covalent JNK inhibitor, JNK-IN-8, suppresses tumor growth in triple-negative breast cancer by activating TFEB and TFE3 mediated lysosome biogenesis and autophagy. Mol Cancer Ther (2022) 21(10):1547–60. doi: 10.1158/1535-7163.MCT-21-1044 35977156

[55] Kojima T Kato K Hara H Takahashi S Muro K Nishina T . Phase II study of BKM120 in patients with advanced esophageal squamous cell carcinoma (EPOC1303). Esophagus (2022) 19(4):702–10. doi: 10.1007/s10388-022-00928-3 PMC9436835 35904643

[56] Shrestha Bhattarai T Shamu T Gorelick AN Chang MT Chakravarty D Gavrila EI . AKT mutant allele-specific activation dictates pharmacologic sensitivities. Nat Commun (2022) 13(1):2111. doi: 10.1038/s41467-022-29638-1 35440569 PMC9018718

[57] Park R Coveler AL Cavalcante L Saeed A . GSK-3β in pancreatic cancer: Spotlight on 9-ING-41, its therapeutic potential and immune modulatory properties. Biology (2021) 10(7):610. doi: 10.3390/biology10070610 34356465 PMC8301062

[58] Salimi A Schroeder KM Schemionek-Reinders M Vieri M Maletzke S Gezer D . Targeting autophagy increases the efficacy of proteasome inhibitor treatment in multiple myeloma by induction of apoptosis and activation of JNK. BMC Cancer (2022) 22(1):735. doi: 10.1186/s12885-022-09775-y 35790913 PMC9258169

[59] Howell SJ Casbard A Carucci M Ingarfield K Butler R Morgan S . Fulvestrant plus capivasertib versus placebo after relapse or progression on an aromatase inhibitor in metastatic, oestrogen receptor-positive, HER2-negative breast cancer (FAKTION): Overall survival, updated progression-free survival, and expanded biomarker analysis from a randomised, phase 2 trial. Lancet Oncol (2022) 23(7):851–64. doi: 10.1016/S1470-2045(22)00284-4 PMC9630162 35671774

[60] Silk AW Saraiya B Groisberg R Chan N Spencer K Girda E . A phase ib dose-escalation study of troriluzole (BHV-4157), an oral glutamatergic signaling modulator, in combination with nivolumab in patients with advanced solid tumors. Eur J Med Res (2022) 27(1):107. doi: 10.1186/s40001-022-00732-w 35780243 PMC9250196

[61] Liu Y Chen C Wang X Sun Y Zhang J Chen J . An epigenetic role of mitochondria in cancer. Cells (2022) 11(16):2518. doi: 10.3390/cells11162518 36010594 PMC9406960

